# Genetik, Epigenetik und Umweltfaktoren der Lebenserwartung – Welche Rolle spielt Nature-versus-Nurture beim Altern?

**DOI:** 10.1007/s00103-024-03873-x

**Published:** 2024-04-18

**Authors:** Holger Bierhoff

**Affiliations:** 1 Institut für Biochemie und Biophysik, Friedrich-Schiller-Universität Jena, Hans-Knöll-Straße 2, 07745 Jena, Deutschland; 2 Leibniz-Institut für Alternsforschung – Fritz-Lipmann-Institut (FLI), Jena, Deutschland

**Keywords:** Alternsforschung, Chronologisches und biologisches Alter, Epigenetik, Epigenetische Marker des Alterns, Geroprotektion, Aging research, Chronological and biological age, Epigenetics, Epigenetic markers of aging, Geroprotection

## Abstract

In Deutschland und weltweit steigt das Durchschnittsalter der Bevölkerung immer weiter an. Mit dieser allgemeinen Zunahme des chronologischen Alters wird der Fokus auf das biologische Alter, d. h. den tatsächlichen Gesundheits- und Fitnesszustand, immer wichtiger. Hier stellt sich die zentrale Frage, inwieweit die altersbedingte Abnahme der Fitness genetisch vorbestimmt oder durch Umweltfaktoren und Lebensstil beeinflussbar ist.

Bei dieser Nature-versus-Nurture-Debatte haben viele epigenetische Studien in der Alternsforschung interessante Einblicke geliefert. In den meisten Modellorganismen geht das Altern mit bestimmten epigenetischen Veränderungen einher, denen unter anderem durch moderate Kalorienreduzierung oder vermehrte körperliche Aktivität entgegengewirkt werden kann. Da sich diese Interventionen auch positiv auf die Lebensspanne und Gesundheit auswirken, scheint die Epigenetik im Mittelpunkt zwischen Umwelteinflüssen und Alternsprozessen zu stehen. Hierfür spricht auch, dass es im Verlauf des Lebens von eineiigen Zwillingen eine epigenetische Drift gibt, die mit der unterschiedlichen Ausprägung von Alterserscheinungen zusammenhängt. Darüber hinaus lässt sich anhand von DNA-Methylierungsmustern das biologische Alter sehr präzise bestimmen, was die Bedeutung der Epigenetik für das Altern weiter untermauert.

Dieser Beitrag gibt eine Übersicht über die Bedeutung von genetischen und epigenetischen Parametern für die Lebenserwartung. Dabei wird ein Augenmerk auf den Möglichkeiten liegen, durch Lebensstil und Umweltfaktoren ein junges Epigenom zu erhalten, um so das biologische Altern zu verlangsamen.

## Abstract (en)

In Germany and worldwide, the average age of the population is continuously rising. With this general increase in chronological age, the focus on biological age, meaning the actual health and fitness status, is becoming more and more important. The key question is to what extent the age-related decline in fitness is genetically predetermined or malleable by environmental factors and lifestyle.

Many epigenetic studies in aging research have provided interesting insights in this nature-versus-nurture debate. In most model organisms, aging is associated with specific epigenetic changes, which can be countered by certain interventions like moderate caloric restriction or increased physical activity. Since these interventions also have positive effects on lifespan and health, epigenetics appears to be the interface between environmental factors and the aging process. This notion is supported by the fact that an epigenetic drift occurs through the life course of identical twins, which is related to the different manifestations of aging symptoms. Furthermore, biological age can be determined with high precision based on DNA methylation patterns, further emphasizing the importance of epigenetics in aging.

This article provides an overview of the importance of genetic and epigenetic parameters for life expectancy. A major focus will be on the possibilities of maintaining a young epigenome through lifestyle and environmental factors, thereby slowing down biological aging.

## Einleitung

Deutschland befindet sich mitten im demografischen Wandel. Die zunehmende Überalterung der Bevölkerung geht mit großen Herausforderungen für die Wirtschaft, das Gesundheitswesen und die Sozialsysteme einher. Zur Meisterung dieser Herausforderungen ist es entscheidend, sowohl die Lebensqualität als auch die Produktivität der Menschen bis ins hohe Lebensalter zu erhalten. Hierbei müssen die Lebenswissenschaften den wichtigen Beitrag leisten, die molekularen und zellulären Alternsprozesse zu entschlüsseln, um aus den Erkenntnissen geroprotektive Maßnahmen ableiten zu können.

Das Verständnis der biologischen Veränderungen, die im Laufe der Zeit auf zellulärer Ebene auftreten und zum Altern beitragen, ist gerade in den letzten 10 Jahren deutlich umfangreicher und detaillierter geworden [1, 2]. Bei diesen Studien hat sich herausgestellt, dass epigenetische Mechanismen maßgeblich am altersbedingten Funktionsverlust der Zelle beteiligt sind.

Darüber hinaus haben genomweite Assoziationsstudien (Genome-wide Association Studies – GWAS) sowie die molekularen Analysen von Progeroid-Syndromen, d. h. genetischen Erkrankungen mit vorzeitigem Auftreten von Alterserscheinungen, Einblicke in die genetischen Faktoren geliefert, die einen signifikanten Einfluss auf die Lebensspanne haben [3, 4]. Obwohl diese Zusammenhänge zwischen Genetik und Lebenserwartung zum grundlegenden Verständnis des biologischen Alterns beitragen, sind sie aufgrund der Unveränderbarkeit des Erbguts für geroprotektive Maßnahmen nicht zugänglich. Dagegen wird die epigenetische Information teilweise durch extrazelluläre Reize und Umweltfaktoren gesteuert. Entsprechend besteht die Möglichkeit, durch geeignete Interventionen der epigenetischen Deregulierung, d. h. dem altersbedingten Verlust und der Umlagerung von Chromatinmodifizierungen, entgegenzuwirken oder vielleicht sogar ein gealtertes Epigenom wieder zu verjüngen.

Der vorliegende Beitrag gibt eine kurze Übersicht über die genetischen und epigenetischen Aspekte des Alterns. Dabei wird der Frage nachgegangen, inwieweit ein langes Leben in guter Gesundheit (Gesundheitsspanne) genetisch determiniert ist oder durch einen auf die Epigenetik wirkenden Lebensstil aktiv gefördert werden kann. Mit der Gegenüberstellung von Genetik und Epigenetik soll die Bedeutung von Nature-versus-Nurture für das Altern beleuchtet werden.

## Einfluss von genetischen Faktoren auf das Altern

Altern im biologischen Sinne geht mit einem Funktionsverlust von Zellen und Organen einher, wodurch wiederum das Auftreten von altersassoziierten Krankheiten begünstigt wird. Diese Alterserscheinungen setzen unterschiedlich stark und früh im individuellen Lebensverlauf ein und haben so einen großen Einfluss auf das Sterbealter. Um die Rolle der Genetik beim Altern zu untersuchen, kann die Lebensspanne an sich als Kriterium verwendet werden oder das Überschreiten einer definierten Altersgrenze (z. B. 90 Jahre), d. h. die Langlebigkeit.

Langlebigkeit tritt oft mit einer Kompression der altersbedingten Morbidität und zudem familiär gehäuft auf [5–7]. Diese Befunde weisen darauf hin, dass der Alternsprozess zu einem gewissen Teil durch erbliche Faktoren bestimmt wird.

Vererbbare genetische Mutationen können in seltenen Fällen auch zu einem Krankheitsbild führen, das einem stark beschleunigten Altern ähnelt. Obwohl diese Progeroid-Syndrome kein physiologisches Altern im Zeitraffer sind, haben ihre Erforschung und die Identifizierung der betroffenen Gene dazu beigetragen, Erkenntnisse über altersbedingte Pathomechanismen und Krankheiten zu gewinnen.

## Genetische Mutationen in Progeroid-Syndromen

Progeroid-Syndrome weisen ähnliche klinische Merkmale auf, aber die zugrunde liegenden Mechanismen können je nach mutiertem Gen variieren [4, 8, 9]. Es lassen sich hinsichtlich der betroffenen zellulären Funktionen bzw. Strukturen 2 Hauptkategorien unterscheiden.

Die erste Gruppe umfasst Syndrome, die durch Veränderungen von Komponenten der Zellkernhülle verursacht werden. Das am besten untersuchte Syndrom dieser Klasse ist das Hutchinson-Gilford-Progerie-Syndrom (HGPS), bei dem in über 90 % der Fälle eine heterozygote Mutation im *LMNA*-Gen vorliegt. *LMNA* codiert für die Proteine Lamin A und Lamin C, die strukturelle Komponenten der Kernhülle sind [10]. Durch die im HGPS auftretende Mutation wird eine fehlgespleißte mRNA gebildet, die zu einer verkürzten Lamin-A-Form, dem sogenannten Progerin, führt. Der Einbau von Progerin destabilisiert die Kernhülle, sodass es zur Störung der Genexpression und der Genomstabilität kommt [11].

Die betroffenen Kinder zeigen ab dem ersten oder zweiten Lebensjahr eine Wachstumsverzögerung und Symptome, die normalerweise im höheren Alter auftreten, wie beispielsweise Haarausfall, Hautverdünnung, Gelenksteifigkeit und Anfälligkeit für Herz-Kreislauf-Erkrankungen. Interessanterweise wurde Progerin auch in Zellen von über 70-jährigen Menschen nachgewiesen, was sich auf eine altersbedingte Veränderung des Spleißens der *LMNA*-mRNA zurückführen lässt [12]. Daher ist anzunehmen, dass das HGPS in der Tat einen Mechanismus des normalen Alterns in verschärfter Form rekapituliert.

In der zweiten Gruppe der Progeroid-Syndrome sind Gene mutiert, die an DNA-Reparaturmechanismen beteiligt sind. Hier ist vor allem das autosomal-rezessiv vererbliche Werner-Syndrom (WS) zu nennen, die verbreitetste Krankheit, bei der Symptome des vorzeitigen Alterns mit Beginn der Pubertät einsetzen [4, 9]. Im WS ist das *WRN*-Gen betroffen, das für eine DNA-Helikase der RecQ-Familie codiert. Das WRN-Protein ist an der Reparatur, Replikation und Transkription der DNA sowie an der Aufrechterhaltung der Telomerlänge und der genomischen Stabilität beteiligt.

Die im WS auftretenden *WRN*-Mutationen bewirken eine Verkürzung oder Instabilität und damit letztendlich einen Funktionsverlust des Proteins. Vorzeitige Alterserscheinungen, wie Ergrauung und Ausfall der Haare, Hautverdünnung und Abnahme des subkutanen Fettgewebes, sind bei WS-erkrankten Personen ab ca. dem 20. Lebensjahr zu beobachten. Dazu kommt das Auftreten von altersassoziierten Krankheiten, wie Katarakten, Diabetes mellitus Typ 2, Arteriosklerose und Osteoporose. Im Gegensatz zum HGPS ist WS mit einem hohen Krebsrisiko verbunden, was sich durch die Störung der DNA-abhängigen Prozesse erklären lässt, an denen die WRN-Helikase beteiligt ist.

In ähnlicher Weise wird ein funktioneller Rückgang der DNA-Reparatursysteme auch beim normalen Altern mit Genominstabilität, Fehlregulation und Zellentartung in Verbindung gebracht [13]. Wie es in diesem Fall aber zur Abnahme der Reparaturkapazität kommt, hat vermutlich vielschichtige Ursachen und wird erst ansatzweise verstanden [2].

## Genvarianten mit Auswirkungen auf die Lebenserwartung

Obwohl die Erforschung von Progeroid-Syndromen wichtige Erkenntnisse über die zellulären Mechanismen des Alterns liefern kann, gibt sie nur begrenzt Hinweise auf die genetischen Faktoren, die die Lebensspanne gesunder Menschen bestimmen. Um dieser Fragestellung nachzugehen, kommen vielmehr GWAS zum Einsatz, die einen Zusammenhang zwischen der Lebenserwartung und bestimmten Genpolymorphismen untersuchen [3].

In diesen Studien werden die Genome von Hochaltrigen analysiert oder es werden Testpersonen in Bezug auf die Lebensspanne der Eltern untersucht. Bei der zweiten Herangehensweise ist somit die Langlebigkeit kein direktes Kriterium, es können aber große Kohorten untersucht werden, wie z. B. die UK-Biobank, die genomische und phänotypische Daten von ca. 500.000 Individuen enthält, wodurch eine hohe Trennschärfe bei den statistischen Tests erzielt werden kann [14]. Dennoch braucht es strenge statistische Signifikanzschwellen und die Bestätigung signifikanter Assoziationen in unabhängigen Replikationen, um falsch-positive Ergebnisse zu vermeiden.

Ein aktueller Übersichtsartikel führt 26 GWAS über das menschliche Altern auf, bei denen insgesamt 55 unabhängige Genloki gefunden wurden, die signifikant mit Langlebigkeit oder der elterlichen Lebensspanne assoziiert sind [15]. Aus dieser relativ großen Zahl an Genen schließen die Autoren und Autorinnen, dass Langlebigkeit im Menschen durch viele Polymorphismen mit mäßigen Ausprägungseffekten bestimmt wird.

Ein Gen, für das der Zusammenhang mit Langlebigkeit in vielen unabhängigen Studien repliziert wurde, ist *APOE*, das für Apolipoprotein E (Apo-E) codiert [3, 16]. Apo‑E ist ein Strukturbestandteil von Lipoproteinen, die für den Transport von Cholesterin und Triglyceriden im Blut zuständig sind. Die 3 Hauptisoformen Apo-E2, Apo-E3 und Apo-E4 sind die Produkte der *ε2-, ε3-* und *ε4-APOE*-Genvarianten. Obwohl sich die Isoformen nur in einer bzw. 2 Aminosäuren unterscheiden, ist das homozygote Vorkommen von *ε4* mit einem erhöhten Risiko für Atherosklerose und Alzheimer-Erkrankung verbunden [17]. Die Begünstigung dieser altersassoziierten Krankheiten erklärt die negative Assoziation des *ε4*-Allels mit Langlebigkeit [18].

Neben *APOE *konnte in genetischen Studien auch für das *FOXO3*-Gen eine Verbindung zur Langlebigkeit reproduzierbar nachgewiesen werden [3, 16, 19, 20]. Der durch *FOXO3* codierte Transkriptionsfaktor ist Teil eines evolutionär konservierten Regulationsnetzwerks, das durch Insulin und den insulinähnlichen Wachstumsfaktor 1 gesteuert wird und dessen Aktivität negativ mit der Lebensspanne korreliert [1, 2]. Die Repression des Signalwegs aktiviert FOXO3 und damit wiederum dessen Zielgene, die die zelluläre Stressresistenz fördern. Interessanterweise sind alle 3 mit Langlebigkeit assoziierten *FOXO3-*Polymorphismen, rs2802292, rs12206094 und rs4946935, in Introns lokalisiert und bewirken eine erhöhte Genexpression [21–23].

Intronische Polymorphismen, deren Assoziation mit der elterlichen Lebensspanne in mehreren Studien entdeckt wurde, befinden sich auch im *CDKN2A/B*-Lokus [3, 15, 20]. Diese Varianten betreffen nicht die proteincodierenden Gene, sondern eine lange nichtcodierende RNA (lncRNA), die als *CDKN2B-AS1* oder *ANRIL* (Antisense Non-coding RNA in the *INK4* Locus) bekannt ist. *CDKN2B-AS1* überlappt in Antisense-Orientierung mit dem Tumorsuppressorgen *CDKN2B/p15*^*INK4B*^ und teilt sich einen bidirektionalen Promotor mit dem *CDKN2A*-Gen, das für die Tumorsuppressoren p16^INK4A^ und p14^ARF^ codiert. Interessanterweise induziert *CDKN2B-AS1* epigenetische Mechanismen, die *CDKN2A/B* reprimieren und somit die Zellproliferation und -entartung fördern [24, 25].

Es ist aber nicht klar, ob diese onkogene Rolle der lncRNA für ihre Assoziation mit der Lebensspanne ausschlaggebend ist, da der *CDKN2B-AS1*-Lokus auch viele Polymorphismen umfasst, die mit atherosklerotischen Herz-Kreislauf-Krankheiten korrelieren. Der Zusammenhang zwischen der lncRNA und Atherosklerose scheint vielschichtig zu sein und über die epigenetische Regulation des *INK4*-Lokus hinauszugehen. Hierfür spricht, dass es eine große Zahl an linearen und zirkulären *CDKN2B-AS1*-Isoformen gibt, die an unterschiedlichen pro- als auch antiatherogenen Mechanismen beteiligt sind [26]. Es ist anzunehmen, dass die *CDKN2B-AS1*-Genvarianten die Synthese der Isoformen beeinflussen und so die Ausprägung dieser Mechanismen steuern.

## Altersbedingte Veränderungen des Epigenoms

Anhand von Zwillingsstudien wurde der genetische Einfluss auf die Lebensspanne auf ca. 20–30 % geschätzt [3]. Eine neuere Arbeit hat dagegen einen Wert von unter 7 % ermittelt und kommt zu dem Schluss, dass die familienbedingte Lebenserwartung stark von assortativer Paarung, d. h. der Wahl eines Partners mit ähnlichem sozioökonomischen Status, abhängt [27]. Ein solch moderater Effekt unserer DNA auf die Lebenserwartung unterstreicht die Bedeutung der Umweltfaktoren, die sich wiederum auf epigenetischer Ebene auswirken können. In der Tat sind Veränderungen in der epigenetischen Landschaft des Zellkerns ein Grundmerkmal des Alterns und betreffen DNA-Methylierung, Histonmodifikationen sowie die generelle Packung des Chromatins [1, 2].

In Bezug auf diese altersassoziierten Veränderungen im Epigenom stellen sich 3 wichtige Fragen:

1. Finden sie gerichtet statt oder handelt es sich um zufällig auftretende Ereignisse?
2. Inwieweit sind sie Auslöser oder Begleiterscheinung des Alternsprozesses?
3. Wie werden sie durch äußere Einflüsse gesteuert?

Ein Meilenstein für die Beantwortung dieser Fragen ist die Entdeckung der epigenetischen Uhren, die DNA-Methylierungsmuster als Surrogatparameter für das biologische Alter etabliert haben.

### Epigenetik als Lebenszeitmesser

Epigenetische Uhren stellen eine innovative Methode zur Abschätzung des altersabhängigen Gesundheitszustands eines Individuums dar. Ihr Funktionsprinzip beruht auf der Analyse von DNA-Methylierung, d. h. der chemischen Modifikation von Cytosinresten in einem Cytosin-Guanin-Dinukleotid (CpG) durch Anheftung einer Methylgruppe. DNA-Methylierung ist in Genomen von Wirbeltieren sehr verbreitet und spielt eine entscheidende Rolle bei der Regulation genetischer Prozesse [28]. Obwohl diese Modifikation als Binärcode (methyliert versus unmethyliert) fungiert, findet man bei der Untersuchung von Zellpopulationen graduelle Unterschiede, je nachdem in welchem Anteil der Zellen ein bestimmtes CpG methyliert vorliegt.

Durch intensive Datenanalysen entdeckte der Genetiker und Biostatistiker Steve Horvath, dass sich diese DNA-Methylierungsmuster mit dem Alter verändern, und entwickelte anhand von 353 CpG-Stellen die erste epigenetische Uhr, die auf ein breites Spektrum von Zelltypen und Geweben des menschlichen Körpers anwendbar ist [29]. Unabhängig davon wurde parallel eine epigenetische Uhr für menschliche Blutzellen beschrieben [30]. Nach Eichung dieser Methylierungsuhren mit dem chronologischen Alter der Testpopulation lässt sich über das individuelle Methylierungsmuster eines Menschen das epigenetische Alter bestimmen. Dieses kann sowohl positiv als auch negativ von den tatsächlichen Lebensjahren abweichen und somit Hinweise auf den Gesundheitszustand einer Person geben.

Basierend auf dieser Pionierleistung wurde eine zweite Generation von epigenetischen Uhren entwickelt, die weitere DNA-Methylierungsmuster mit Aussagekraft über altersbezogene Biomarker (z. B. Entzündungsmarker, Nierenfunktion und Blutzuckerwerte) und Krankheitsrisiken (z. B. Tabakrauchexposition) einbeziehen. Entsprechend nutzen diese als PhenoAge und GrimAge bezeichneten Uhren mehr CpG-Stellen (513 bzw. 1030 CpGs) und können die Lebenserwartung und verbleibende Lebensspanne in Gesundheit vorhersagen [31, 32].

Die verbesserte Vorhersagekraft dieser Uhren durch die Hinzunahmen gesundheitsrelevanter Parameter unterstreicht den starken Einfluss der Umwelt auf den biologischen bzw. epigenetischen Alternsprozess. Andererseits spiegelt die epigenetische Altersmessung die menschlichen Entwicklungsphasen wider, indem sie in der frühen Embryogenese mit dem Wert 0 startet, dann exponentiell ansteigt und ab der Pubertät zu einem linearen Verlauf abflacht. Somit wird das Altern des Epigenoms zum einen durch eine intrinsische Komponente, zum anderen durch äußere Faktoren gesteuert, die sich auf den Metabolismus, die Integrität der DNA und die Chronobiologie auswirken [33].

## Beschleunigtes Altern durch epigenetische Deregulierung

Durch das Chromatin wird nicht nur die Funktion des Genoms in einer Zelle kontrolliert, sondern die epigenetische Information wird auch an die Tochterzellen weitergegeben. Hierbei treten im Laufe der Zeit Fehler auf, die zu einer epigenetischen Deregulierung führen. Beispielsweise wird die DNA-Methylierung nicht vollständig kopiert, sodass es mit zunehmender Anzahl an Zellteilungen zu einem Verlust an dieser Modifikation kommt. Dieser Effekt trägt teilweise zum Ticken der epigenetischen Uhren bei, bei denen es allerdings auch CpGs gibt, die mit dem Alter verstärkt methyliert werden [33]. Da die DNA-Methylierung eine wichtige Rolle bei der Stilllegung von transponierbaren DNA-Elementen, wie z. B. endogenen Retroviren, spielt, kann ihr Verlust zu einer Aktivierung dieser Elemente führen, was wiederum die Funktion und Stabilität des Genoms beeinträchtigen kann [34].

Neben der Demethylierung der DNA kommt es mit fortschreitenden Zellteilungen auch zur Hemmung der Histonsynthese. Der Auslöser hierfür sind chronische DNA-Schäden, die durch die Verkürzung der Telomere entstehen [35]. Da Histone die als Nukleosomen bezeichneten Proteinkomplexe bilden, um die die DNA gewunden ist, wird durch ihren Verlust die Packung des Chromatins aufgelockert. Dies betrifft vor allem das stark kondensierte Heterochromatin, das für die Genrepression und den Schutz der Chromosomenintegrität zuständig ist, und trägt somit zur irregulären Genaktivität und Genominstabilität bei [36].

Darüber hinaus legt eine aktuelle Studie nahe, dass mit dem Alter die Nukleosomendichte von Genen vermindert ist, sodass sie schneller, aber ungenauer abgelesen werden. Erstaunlicherweise konnte dieser Defekt in Fruchtfliegen durch Überexpression von Histonen wieder behoben werden, was zu einer verlängerten Lebensspanne führte [37].

Eine Abnahme der Genauigkeit in der epigenetischen Regulation mit dem Alter wird auch als epigenetische Drift bezeichnet, die sich aber nur zum Teil über die mitotische Aktivität der Zellen erklären lässt. Eine kürzlich veröffentlichte Studie, die von dem bekannten Alternsforscher David Sinclair geleitet wurde, konnte die zentrale Bedeutung von DNA-Schäden, insbesondere von DNA-Doppelstrangbrüchen (DSB), für die epigenetische Drift nachweisen [38]. In dieser Arbeit wurden Mäuse genetisch so verändert, dass in ihren Zellen DSB für eine bestimmte Zeit und an definierten Stellen im Genom induziert werden konnten. Aufgrund der zeitlichen Begrenzung und der geringen Anzahl an DSB konnte die DNA wieder fehlerfrei repariert werden, sodass keine Mutationen entstanden. Molekulare Analysen zeigten aber Veränderungen im Epigenom, die sowohl die DNA-Methylierung und die Histonmodifikationen als auch die übergeordnete Chromatinorganisation betrafen.

Messungen mit 2 mausspezifischen epigenetischen Uhren ergaben ein um 50 % beschleunigtes Altern, das auch in vielen Tests phänotypisch nachweisbar war. Folglich kommt es durch DNA-Schädigungen und den anschließenden Reparaturprozess zu einer Umverteilung von epigenetischer Information, die nicht mehr vollständig in den Ausgangszustand zurückversetzt werden kann. Damit gehen Zelltyp-spezifische epigenetische Muster verloren, was zu einem Funktionsverlust führt [38]. Die Ergebnisse machen deutlich, dass die Geschwindigkeit, mit der die epigenetische Präzision verloren geht, als Schrittmacher des biologischen Alterns fungiert. Allerdings ist anzumerken, dass die unnatürliche DNA-Schädigung in dem Mausmodell das Altern nicht nur über epigenetische Mechanismen beschleunigt haben könnte.

## Verzögerung des epigenetischen Alterns durch Lebensstil und Umweltfaktoren

Mit der Erkenntnis, dass die Erosion des Epigenoms die Lebens- und Gesundheitsspanne begrenzt, stellt sich die Frage nach geeigneten Maßnahmen, um diesem Trend entgegenzuwirken. Hierfür haben Sinclair und sein Team in dem oben erwähnten Tiermodell eine Methode angewendet, die auf der von dem Nobelpreisträger Shin’ya Yamanaka entdeckten Reprogrammierung von somatischen Zellen zu induzierten pluripotenten Stammzellen beruht [39].

Aus dem ursprünglichen Yamanaka-Cocktail, der die 4 Transkriptionsfaktoren Oct4, Sox2, c‑Myc und Klf4 enthält, wurde c‑Myc entfernt, da es für die initiale Reprogrammierung nicht notwendig ist [40]. Durch Expression der übrigen Faktoren nach Induktion von DSB wurden die hervorgerufenen Aberrationen im Epigenom teilweise korrigiert und somit die Mäuse biologisch wieder verjüngt [38]. In einer anderen Studie wurde durch diese partielle Reprogrammierung in einem Mausmodell des Glaukoms, d. h. einer altersassoziierten Erkrankung des Sehnervs, eine Verjüngung der entsprechenden Neuronen und damit eine Wiedererlangung des Sehvermögens erzielt [41].

Die Umkehrung des epigenetischen Alterns durch Yamanaka-Faktoren ist zwar verblüffend, aber im Hinblick auf Geroprotektion beim Menschen nicht einsetzbar. Daher besteht ein großes Interesse daran zu verstehen, wie sich der Lebensstil und Umweltfaktoren auf das biologische Alter auswirken. Hier spielen vor allem körperliche Aktivität und Ernährung eine Rolle.

Hinsichtlich der körperlichen Aktivität wirken sowohl aerobe Sportarten und Widerstandstraining als auch entspannungsfokussierte Übungen wie Yoga altersbedingten Änderungen der DNA-Methylierung entgegen [42]. Zudem weist eine spezielle epigenetische Uhr, DNAmFitAge, auf einen geroprotektiven Effekt durch erhöhte physiologische Fitness hin [43]. Allerdings sind die molekularen Mechanismen, über die die körperliche Aktivität das Epigenom moduliert, noch nicht so gut verstanden wie bei der Ernährung. Hier hat sich herausgestellt, dass eine Verringerung der Kalorienzufuhr zwischen 10 % und 40 % das biologische Altern verzögert – eine Beobachtung, die praktisch in allen Modellorganismen bestätigt werden konnte [42].

Besonders beeindruckend ist eine Studie in Rhesusaffen, bei denen eine langjährige Kalorienreduzierung um 30 % durchgeführt wurde. Diese Tiere hatten ein chronologisches Durchschnittsalter von 27 Jahren, während das anhand von DNA-Methylierung bestimmte biologische Alter bei 20 Jahren lag [44]. Trotz dieser eindeutigen experimentellen Datenlage ist eine Geroprotektion durch verminderte Kalorienzufuhr beim Menschen noch nicht eindeutig geklärt. Eine großangelegte Studie (CALERIE 2), die über 2 Jahre eine 25 %ige Kalorienreduzierung bei normalgewichtigen Menschen verfolgte, fand keine signifikante Verminderung des biologischen Alters in PhenoAge- und GrimAge-Messungen, konnte aber eine Verzögerung des Alternsprozesses anhand einer weiteren epigenetischen Uhr, dem DunedinPACE-DNAm-Algorithmus, nachweisen [45].

Ein Problem der Kalorienreduzierung in der Ernährung des Menschen ist die langfristige und konsequente Durchführung einer solchen Intervention. Entsprechend wird auch nach Nähr- und Wirkstoffen gesucht, die den Effekt der Kalorienreduzierung imitieren können. Die Forschung an diesen sogenannten Caloric Restriction Mimetics hat sich in den letzten Jahren stark ausgeweitet und umfasst sehr unterschiedliche Substanzen, wie etwa das aus dem Rotwein bekannte Resveratrol, das in Weizenkeimen angereicherte Spermidin oder das Immunsuppressivum Rapamycin [46].

Ein vielversprechender Wirkstoff ist Metformin, das als Arzneimittel bei Typ-2-Diabetes eingesetzt wird. In seiner medizinischen Wirkung drosselt Metformin die Neubildung von Glukose in der Leber, was den Blutzuckerspiegel senkt. Dagegen ist der geroprotektive Effekt von Metformin noch nicht genau aufgeklärt worden, obwohl er in zahlreichen Tiermodellen nachgewiesen werden konnte [47]. Um beim Menschen einen tieferen Einblick in die geroprotektiven Effekte von Metformin zu erlangen, ist eine klinische Studie („Targeting Aging with Metformin“, TAME) mit 3000 Probanden geplant und kurz vor der Umsetzung (https://www.afar.org/tame-trial). TAME wird die erste von der US-Behörde für Lebens- und Arzneimittel zugelassene Studie sein, die die medikamentöse Behandlung des biologischen Alterns erforscht.

In einer viel kleineren Studie mit nur 9 männlichen Probanden im Alter zwischen 51 und 65 Jahren wurde Metformin in Kombination mit rekombinantem menschlichen Wachstumshormon (rhGH) und Dehydroepiandrosteron (DHEA) eingesetzt, um die altersbedingte Thymusrückbildung zu revertieren [48]. Allerdings war hier rhGH der eigentliche Wirkstoff für die Thymusregeneration, während Metformin und DHEA dem Ansteigen des Glukosespiegels, das als rhGH-Nebenwirkung auftritt, entgegenwirken sollten. Durch die einjährige Behandlung konnte nicht nur die Funktion des Thymus stimuliert werden, sondern es wurde auch das durchschnittliche epigenetische Alter um 2,5 Jahre zurücksetzt [48]. Aufgrund der sehr geringen Teilnehmeranzahl der Studie sind diese Ergebnisse aber kritisch zu betrachten; der Ausgang einer größer angelegten Nachfolgestudie (TRIIM‑X; https://clinicaltrials.gov/study/NCT04375657) bleibt abzuwarten.

## Fazit

Das biologische Altern ist ein komplexes Phänomen, dessen molekulare Grundlagen intensiv erforscht werden. Die immer besser und günstiger werdenden Techniken für Hochdurchsatzsequenzierung ermöglichen es, die genetischen Grundlagen des Alterns an einer großen Anzahl an Testpersonen zu untersuchen. Außerdem tragen auch neue statistische Verfahren, wie multivariate GWAS, zur Identifizierung altersrelevanter Genpolymorphismen bei [49–51].

Wie wir altern, wird zu einem (geringen) Teil von unseren Genen vorbestimmt und hängt zudem von der Jungerhaltung des Epigenoms ab. Aber auch andere zelluläre Prozesse, die von der Umwelt beeinflusst werden können, spielen beim Erreichen einer langen Gesundheitsspanne eine wichtige Rolle. Diese Prozesse werden durch moderne Hochdurchsatzverfahren, wie Proteomics oder Metabolomics, immer besser verstanden [52].

Insgesamt zeigt dieses Wissen Möglichkeiten auf, der biologischen Alterung aktiv entgegenzuwirken. Hierfür ist die einfache Maxime „weniger Essen und mehr Bewegung“ vermutlich nicht besonders attraktiv, dafür aber wirksam. Für ein gesundes Altern spielen neben Ernährung und Bewegung noch weitere Lebensgewohnheiten und Umweltfaktoren, wie ausreichender Schlaf, geringer psychischer Stress und ein positives soziales Umfeld, eine wichtige Rolle [53]. Wie das Zusammenspiel dieser Faktoren auf das Epigenom wirkt, ist eine spannende Frage, die in der Zukunft mit den epigenetischen Uhren weiter aufgeschlüsselt werden kann. Diese Uhren werden auch hilfreich für die Bewertung des geroprotektiven Potenzials einzelner Substanzen oder Substanzkombinationen sein. Aufgrund der Vielschichtigkeit des Alternsprozesses ist allerdings davon auszugehen, dass solche Substanzen bei der lebenslangen Gesunderhaltung bestenfalls unterstützend wirken können, aber kein Ersatz für einen gesunden Lebensstil sein werden.

## References

[1] Lopez-Otin C, Blasco MA, Partridge L, Serrano M, Kroemer G (2013). The hallmarks of aging. Cell.

[2] Lopez-Otin C, Blasco MA, Partridge L, Serrano M, Kroemer G (2023). Hallmarks of aging: An expanding universe. Cell.

[3] Melzer D, Pilling LC, Ferrucci L (2020). The genetics of human ageing. Nat Rev Genet.

[4] Schnabel F, Kornak U, Wollnik B (2021). Premature aging disorders: A clinical and genetic compendium. Clin Genet.

[5] Sebastiani P, Perls TT (2012). The genetics of extreme longevity: lessons from the new England centenarian study. Front Genet.

[6] van den Berg N, Beekman M, Smith KR, Janssens A, Slagboom PE (2017). Historical demography and longevity genetics: Back to the future. Ageing Res Rev.

[7] van den Berg N, Rodriguez-Girondo M, van Dijk IK (2019). Longevity defined as top 10 % survivors and beyond is transmitted as a quantitative genetic trait. Nat Commun.

[8] Carrero D, Soria-Valles C, Lopez-Otin C (2016). Hallmarks of progeroid syndromes: lessons from mice and reprogrammed cells. Dis Model Mech.

[9] Puzianowska-Kuznicka M, Kuznicki J (2005). Genetic alterations in accelerated ageing syndromes. Int J Biochem Cell Biol.

[10] Fisher DZ, Chaudhary N, Blobel G (1986). cDNA sequencing of nuclear lamins A and C reveals primary and secondary structural homology to intermediate filament proteins. Proc Natl Acad Sci U S A.

[11] Gonzalo S, Kreienkamp R, Askjaer P (2017). Hutchinson-Gilford Progeria Syndrome: A premature aging disease caused by LMNA gene mutations. Ageing Res Rev.

[12] Scaffidi P, Misteli T (2006). Lamin A-dependent nuclear defects in human aging. Science.

[13] Vijg J, Suh Y (2013). Genome instability and aging. Annu Rev Physiol.

[14] Bycroft C, Freeman C, Petkova D (2018). The UK Biobank resource with deep phenotyping and genomic data. Nature.

[15] Zhang ZD, Milman S, Lin JR (2020). Genetics of extreme human longevity to guide drug discovery for healthy ageing. Nat Metab.

[16] Smulders L, Deelen J (2023). Genetics of human longevity: From variants to genes to pathways. J Intern Med.

[17] Abondio P, Sazzini M, Garagnani P (2019). The Genetic Variability of APOE in Different Human Populations and Its Implications for Longevity. Genes (basel).

[18] Sebastiani P, Gurinovich A, Nygaard M (2019). APOE Alleles and Extreme Human Longevity. J Gerontol A Biol Sci Med Sci.

[19] Broer L, Buchman AS, Deelen J (2015). GWAS of longevity in CHARGE consortium confirms APOE and FOXO3 candidacy. J Gerontol A Biol Sci Med Sci.

[20] Deelen J, Evans DS, Arking DE (2019). A meta-analysis of genome-wide association studies identifies multiple longevity genes. Nat Commun.

[21] Flachsbart F, Dose J, Gentschew L (2017). Identification and characterization of two functional variants in the human longevity gene FOXO3. Nat Commun.

[22] Grossi V, Forte G, Sanese P (2018). The longevity SNP rs2802292 uncovered: HSF1 activates stress-dependent expression of FOXO3 through an intronic enhancer. Nucleic Acids Res.

[23] Hjazi A, Ghaffar E, Asghar W (2023). CDKN2B-AS1 as a novel therapeutic target in cancer: Mechanism and clinical perspective. Biochem Pharmacol.

[24] Kotake Y, Nakagawa T, Kitagawa K (2011). Long non-coding RNA ANRIL is required for the PRC2 recruitment to and silencing of p15(INK4B) tumor suppressor gene. Oncogene.

[25] Yap KL, Li S, Munoz-Cabello AM (2010). Molecular interplay of the noncoding RNA ANRIL and methylated histone H3 lysine 27 by polycomb CBX7 in transcriptional silencing of INK4a. Mol Cell.

[26] Holdt LM, Teupser D (2018). Long Noncoding RNA ANRIL: Lnc-ing Genetic Variation at the Chromosome 9p21 Locus to Molecular Mechanisms of Atherosclerosis. Front Cardiovasc Med.

[27] Ruby JG, Wright KM, Rand KA (2018). Estimates of the Heritability of Human Longevity Are Substantially Inflated due to Assortative Mating. Nat Genet.

[28] Schubeler D (2015). Function and information content of DNA methylation. Nature.

[29] Horvath S (2013). DNA methylation age of human tissues and cell types. Genome Biol.

[30] Hannum G, Guinney J, Zhao L (2013). Genome-wide methylation profiles reveal quantitative views of human aging rates. Mol Cell.

[31] Levine ME, Lu AT, Quach A (2018). An epigenetic biomarker of aging for lifespan and healthspan. Aging (albany Ny).

[32] Lu AT, Binder AM, Zhang J (2022). DNA methylation GrimAge version 2. Aging (albany Ny).

[33] Seale K, Horvath S, Teschendorff A, Eynon N, Voisin S (2022). Making sense of the ageing methylome. Nat Rev Genet.

[34] Johnstone SE, Gladyshev VN, Aryee MJ, Bernstein BE (2022). Epigenetic clocks, aging, and cancer. Science.

[35] O’Sullivan RJ, Kubicek S, Schreiber SL, Karlseder J (2010). Reduced histone biosynthesis and chromatin changes arising from a damage signal at telomeres. Nat Struct Mol Biol.

[36] Pal S, Tyler JK (2016). Epigenetics and aging. Sci Adv.

[37] Debes C, Papadakis A, Gronke S (2023). Ageing-associated changes in transcriptional elongation influence longevity. Nature.

[38] Yang JH, Hayano M, Griffin PT (2023). Loss of epigenetic information as a cause of mammalian aging. Cell.

[39] Takahashi K, Yamanaka S (2006). Induction of pluripotent stem cells from mouse embryonic and adult fibroblast cultures by defined factors. Cell.

[40] Rand TA, Sutou K, Tanabe K (2018). MYC Releases Early Reprogrammed Human Cells from Proliferation Pause via Retinoblastoma Protein Inhibition. Cell Rep.

[41] Lu Y, Brommer B, Tian X (2020). Reprogramming to recover youthful epigenetic information and restore vision. Nature.

[42] Rajado AT, Silva N, Esteves F (2023). How can we modulate aging through nutrition and physical exercise? An epigenetic approach. Aging (albany Ny).

[43] McGreevy KM, Radak Z, Torma F (2023). DNAmFitAge: biological age indicator incorporating physical fitness. Aging (albany Ny).

[44] Maegawa S, Lu Y, Tahara T (2017). Caloric restriction delays age-related methylation drift. Nat Commun.

[45] Waziry R, Ryan CP, Corcoran DL (2023). Effect of long-term caloric restriction on DNA methylation measures of biological aging in healthy adults from the CALERIE trial. Nat Aging.

[46] Madeo F, Carmona-Gutierrez D, Hofer SJ, Kroemer G (2019). Caloric Restriction Mimetics against Age-Associated Disease: Targets, Mechanisms, and Therapeutic Potential. Cell Metab.

[47] Kulkarni AS, Gubbi S, Barzilai N (2020). Benefits of Metformin in Attenuating the Hallmarks of Aging. Cell Metab.

[48] Fahy GM, Brooke RT, Watson JP (2019). Reversal of epigenetic aging and immunosenescent trends in humans. Aging Cell.

[49] Rosoff DB, Mavromatis LA, Bell AS (2023). Multivariate genome-wide analysis of aging-related traits identifies novel loci and new drug targets for healthy aging. Nat Aging.

[50] Timmers P, Tiys ES, Sakaue S (2022). Mendelian randomization of genetically independent aging phenotypes identifies LPA and VCAM1 as biological targets for human aging. Nat Aging.

[51] Timmers P, Wilson JF, Joshi PK, Deelen J (2020). Multivariate genomic scan implicates novel loci and haem metabolism in human ageing. Nat Commun.

[52] Rutledge J, Oh H, Wyss-Coray T (2022). Measuring biological age using omics data. Nat Rev Genet.

[53] Castruita PA, Piña-Escudero SD, Rentería ME, Yokoyama JS (2022). Genetic, Social, and Lifestyle Drivers of Healthy Aging and Longevity. Curr Genet Med Rep.

